# A bacterial tyrosine phosphatase modulates cell proliferation through targeting RGCC

**DOI:** 10.1371/journal.ppat.1009598

**Published:** 2021-05-20

**Authors:** Chengcheng Liu, Kendall Stocke, Zackary R. Fitzsimonds, Lan Yakoumatos, Daniel P. Miller, Richard J. Lamont

**Affiliations:** Department of Oral Immunology and Infectious Diseases, University of Louisville School of Dentistry, Louisville, KY, United States of America; University of California, Davis, UNITED STATES

## Abstract

Tyrosine phosphatases are often weaponized by bacteria colonizing mucosal barriers to manipulate host cell signal transduction pathways. *Porphyromonas gingivalis* is a periodontal pathogen and emerging oncopathogen which interferes with gingival epithelial cell proliferation and migration, and induces a partial epithelial mesenchymal transition. *P*. *gingivalis* produces two tyrosine phosphatases, and we show here that the low molecular weight tyrosine phosphatase, Ltp1, is secreted within gingival epithelial cells and translocates to the nucleus. An *ltp1* mutant of *P*. *gingivalis* showed a diminished ability to induce epithelial cell migration and proliferation. Ltp1 was also required for the transcriptional upregulation of Regulator of Growth and Cell Cycle (RGCC), one of the most differentially expressed genes in epithelial cells resulting from *P*. *gingivalis* infection. A phosphoarray and siRNA showed that *P*. *gingivalis* controlled RGCC expression through Akt, which was activated by phosphorylation on S473. Akt activation is opposed by PTEN, and *P*. *gingivalis* decreased the amount of PTEN in epithelial cells. Ectopically expressed Ltp1 bound to PTEN, and reduced phosphorylation of PTEN at Y336 which controls proteasomal degradation. Ltp-1 induced loss of PTEN stability was prevented by chemical inhibition of the proteasome. Knockdown of RGCC suppressed upregulation of Zeb2 and mesenchymal markers by *P*. *gingivalis*. RGCC inhibition was also accompanied by a reduction in production of the proinflammatory cytokine IL-6 in response to *P*. *gingivalis*. Elevated IL-6 levels can contribute to periodontal destruction, and the *ltp1* mutant of *P*. *gingivalis* incited less bone loss compared to the parental strain in a murine model of periodontal disease. These results show that *P*. *gingivalis* can deliver Ltp1 within gingival epithelial cells, and establish PTEN as the target for Ltp1 phosphatase activity. Disruption of the Akt1/RGCC signaling axis by Ltp1 facilitates *P*. *gingivalis*-induced increases in epithelial cell migration, proliferation, EMT and inflammatory cytokine production.

## Author summary

Bacteria colonizing the oral cavity can induce inflammatory destruction of the periodontal tissues, and are increasingly associated with oral squamous cell carcinoma. *P*. *gingivalis*, a major periodontal pathogen, can subvert epithelial pathways that control important physiological processes relating to innate immunity and cell fate; however, little is known about the effector molecules. Here we show that *P*. *gingivalis* can deliver a tyrosine phosphatase, Ltp1, within epithelial cells, and Ltp1 phosphatase activity destabilizes PTEN, a negative regulator of Akt1 signaling. The production of RGCC is thus increased and this leads to increased epithelial cell migration, proliferation, a partial mesenchymal phenotype and inflammatory cytokine production. Ltp1 phosphatase activity thus provides a mechanistic basis for a number of *P*. *gingivalis* properties that contribute to disease. Indeed, an Ltp1-deficient mutant was less pathogenic in a murine model of periodontitis. These results contribute to deciphering the pathophysiological events that underlie oral bacterial diseases that initiate at mucosal barriers.

## Introduction

*Porphyromonas gingivalis* is an indigenous constituent of the polymicrobial communities that colonize oral mucosal barriers [1,2]. As a keystone pathogen, *P*. *gingivalis* can elevate community pathogenicity, or nososymbiocity, and induce dysbiotic host responses which contribute to periodontal diseases [3]. *P*. *gingivalis* expresses an array of virulence factors which enable colonization and survival in the oral cavity, community interactions, and disruption of tissue and immune homeostasis. These include fimbrial adhesins, exopolysaccharides, proteolytic enzymes, kinases and phosphatases [2,4]. In the latter category, a serine phosphatase (SerB) produced by *P*. *gingivalis* is released within epithelial cells and dephosphorylates the serine 538 residue of the p65 subunit of NF-κB. The formation of active p65-p65 homodimers is thus prevented, and consequently synthesis of neutrophil chemokine IL-8 is suppressed [5]. *P*. *gingivalis* also produces tyrosine phosphatases: the low molecular weight tyrosine phosphatase, Ltp1 [6], and the polymerase and histidinol phosphatase, Php1 [7]. Both enzymes can dephosphorylate the tyrosine kinase Ptk1, and this tyrosine kinase-phosphatase axis controls exopolysaccharide production, community development with the partner species *Streptococcus gordonii*, and pathogenicity of *P*. *gingivalis* in an animal model of periodontal disease [7,8]. However, while tyrosine residues on host proteins can be dephosphorylated by *P*. *gingivalis* [9], the extent to which Ltp1 or Php1 are active intracellularly within epithelial cells has yet to be determined.

*P*. *gingivalis* is also emerging as an oncopathogen, and elevated numbers of the organism on mucosal surfaces are associated with oral squamous cell carcinoma (OSCC) [10–13]. Infection of epithelial cells with *P*. *gingivalis* impacts a number of properties related to cell fate which could contribute to tumorigenesis. Cell proliferation is increased by modulation of cyclin/CDK (cyclin-dependent kinase) activity, and by a lower level of the p53 tumor suppressor protein[14,15]. The gingipain proteases of *P*. *gingivalis* may also contribute to cell proliferation through proteolytic degradation and activation of β-catenin. Nuclear translocation and accumulation of active β-catenin fragments drives the activity of the β-catenin-dependent, pro-proliferative TCF/LEF promoter [16]. Activation of β-catenin by *P*. *gingivalis* also leads to increased migration and a partial epithelial mesenchymal transition (EMT), through upregulation of the transcription factor Zeb2 [17]. Indeed, expression and activity of many proliferative and migration factors is enhanced by *P*. *gingivalis* including FAK, matrix metalloproteinases, Stat3, and FOXO1 [18–23].

Specific patterns of protein (de)phosphorylation, usually on serine/threonine or tyrosine residues, constitute a major means of control of epithelial cell signaling pathways which regulate cell fate [24]. Phosphorylation can modulate protein–protein interactions, control protein stability, and regulate function and localization. Finely tuned activity of specific kinases and phosphatases is thus essential for normal epithelial cell function [25]. Low molecular weight protein tyrosine phosphatases (LMWPTPs) are emerging as an important signaling hub in cancer, and increased LMWPTP expression is positively correlated with clinical and pathological progression [26–28]. LMWPTPs are associated with resistance to chemotherapeutics and confer survival and growth advantages to tumor cells by promoting the Warburg effect, together with expression of SOD and catalase [29]. LMWPTP activity can also contribute to metastasis, although the phosphatase targets are unknown [27].

In this study we sought to examine the accessibility of the *P*. *gingivalis* Ltp1 tyrosine phosphatase within epithelial cells, identify potential target proteins, and characterize the role of Ltp1 in epithelial cell proliferation and migration.

## Results

### Role of Ltp1 in epithelial cell proliferation and migration

**Fig 1:**
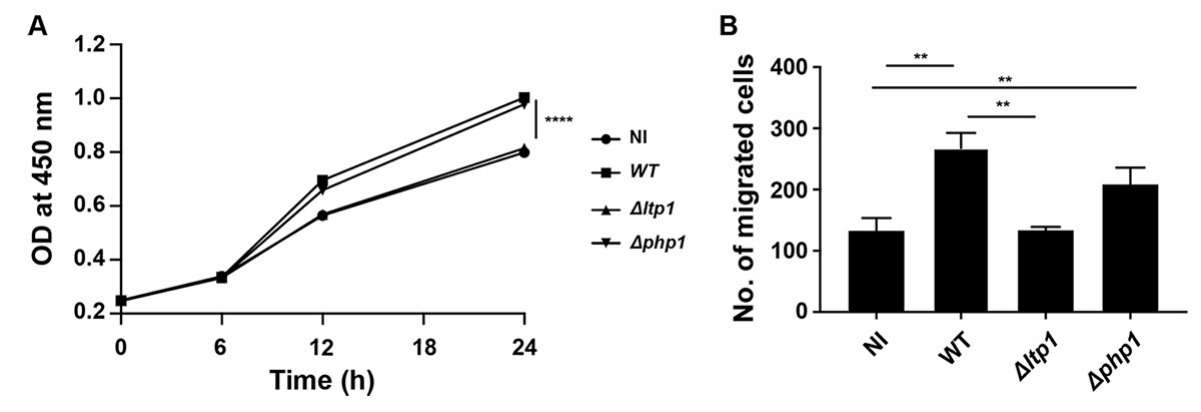
Ltp1 is required for proliferation and migration induced by *P*. *gingivalis* in TIGKs. A) BrdU proliferation ELISA of TIGKs challenged with *P*. *gingivalis* WT, Δ*ltp1*, Δ*php1*, or left uninfected (NI). Absorbance at 450 nm was measured over the times indicated and the results are representative of 3 biological replicates. B) Quantitative analysis of TIGK migration through Matrigel-coated transwells. TIGK cells were challenged with *P*. *gingivalis* WT, Δ*ltp1*, Δ*php1*, or left uninfected (NI). Data are the mean with SD number of cells invading through inserts coated with Matrigel, and are representative of 3 biological replicates. ** p< 0.01, **** p < 0.001.

The involvement of Ltp1 in *P*. *gingivalis*-induced migration and proliferation of gingival epithelial cells was investigated using isogenic mutants. As shown in Fig 1A, disruption of the *ltp1* gene abrogated the ability of *P*. *gingivalis* to increase proliferation of TIGK cells. In contrast, loss of the Php1 phosphatase had no effect on the ability of *P*. *gingivalis* to stimulate TIGK proliferation. Similarly, the Δ*ltp1* strain, but not the Δ*php1* strain, was unable to induce migration of TIGKs (Fig 1B). Thus, Ltp1 is required by *P*. *gingivalis* to both elevate proliferation and migration of TIGK cells.

### Ltp1 is secreted and functional within epithelial cells

**Fig 2:**
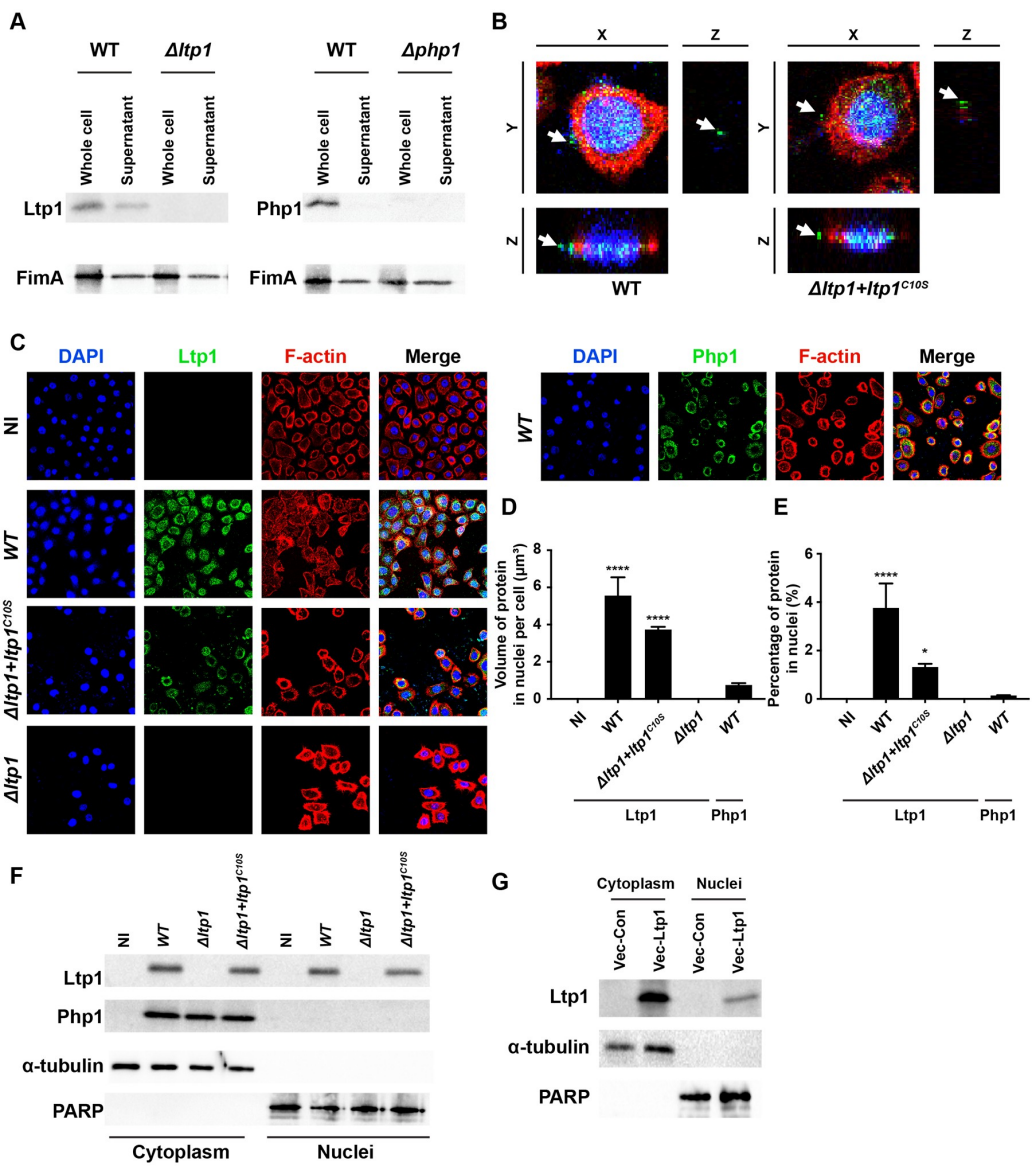
Ltp1 is secreted by *P*. *gingivalis* and co-localizes with the nucleus. A) Western blots of whole-cell lysates or culture supernatants of *P*. *gingivalis* WT, Δ*ltp1*, or Δ*php1*, probed with antibodies to Ltp1, Php1, or FimA as a control for a secreted protein. B). Fluorescent confocal microscopy of TIGK cells challenged with *P*. *gingivalis* WT or Δ*ltp1*+*ltp1*^C10S^. Cells were probed with Ltp1 antibodies and Alexa Fluor 488 secondary antibody (green). Actin (red) was stained with Texas Red-phalloidin, and TIGK and *P*. *gingivalis* DNA stained with DAPI (blue). Cells were imaged at magnification ×63, and shown are digitally reconstructed images (x-z section; and z-projection of x-y sections) generated with Volocity. Arrows indicate areas of secreted Ltp1. C) Fluorescent confocal microscopy of TIGK cells challenged with *P*. *gingivalis* WT, Δ*ltp1*, Δ*php1*, Δ*ltp1*+*ltp1*^C10S^, or left uninfected (NI). Cells were probed with Ltp1 or Php1 antibodies as indicated, and Alexa Fluor 488 secondary antibody (green). Actin (red) was stained with Texas Red-phalloidin, and nuclei (blue) stained with DAPI. Cells were imaged at magnification ×63, and shown are projections of z-stacks generated with Volocity. D) Volume and E) Percent co-localization of Ltp1 or Php1 in the nuclear region of the images in (C) * p< 0.05, **** p < 0.001 compared to NI. F) Western blots of cytoplasmic or nuclear fractions of TIGKs challenged with *P*. *gingivalis* WT, Δ*ltp1*, Δ*php1*, Δ*ltp1*+*ltp1*^C10S^, or left uninfected (NI). Blots were probed with antibodies to Ltp1, Php1, α-tubulin (cytoplasmic control) or PARP (nuclear control). G) Western blots of cytoplasmic or nuclear fractions of TIGKs transiently transfected with Ltp1 (Vec-Ltp1), or empty vector control (Vec-Con). Blots were probed with antibodies to Ltp1, α-tubulin (cytoplasmic control) or PARP (nuclear control).

To assess the mechanism of action of Ltp1, we first determined whether Ltp1 is released from *P*. *gingivalis* into the bacterial extracellular milieu. Probing culture supernatants of *P*. *gingivalis* by immunoblotting (Fig 2A) revealed the presence of Ltp1 extracellular to the bacterial cells. Specificity was corroborated by the absence of the corresponding band in bacterial culture supernatants from the *ltp1* mutant strain. By contrast, secretion of Php1 was not detected, whereas FimA was detected in the supernatants of WT and mutant strains. To confirm that Ltp1 is also produced extracellular to the bacteria in the context of an epithelial cell infection, confocal microscopy was performed. Fig 2B shows Ltp1 clearly distinct from DAPI stained *P*. *gingivalis*. Ltp1 was also produced extracellular to the bacterial cells by the Δ*ltp1* mutant strain complemented with catalytically dead enzyme, C10SLtp1 [6], establishing that secretion does not depend on phosphatase activity. Next, we examined the location of secreted Ltp1 within TIGK cells. As shown in Fig 2C–2E, Ltp1 localized to the nuclear area, and this effect was also independent of phosphatase activity. In contrast, Php1 showed no co-localization with the DAPI-stained nucleus. Confirmation of nuclear localization of Ltp1 was obtained by fractionation of infected cells and immunoblotting. Fig 2F shows that Ltp1 could be detected in the cytoplasmic and nuclear fractions of TIGKs infected with *P*. *gingivalis* parental and C10SLtp1-expressing strains. Php1, however, was only detected in the cytoplasmic fraction. Moreover, ectopic expression of Ltp1 resulted in translocation of the phosphatase to the nucleus (Fig 2G). Collectively, these results show that Ltp1 is released extracellularly by *P*. *gingivalis*, and in TIGKs cells is translocated to the nucleus.

### Eukaryotic targets of Ltp1

**Fig 3:**
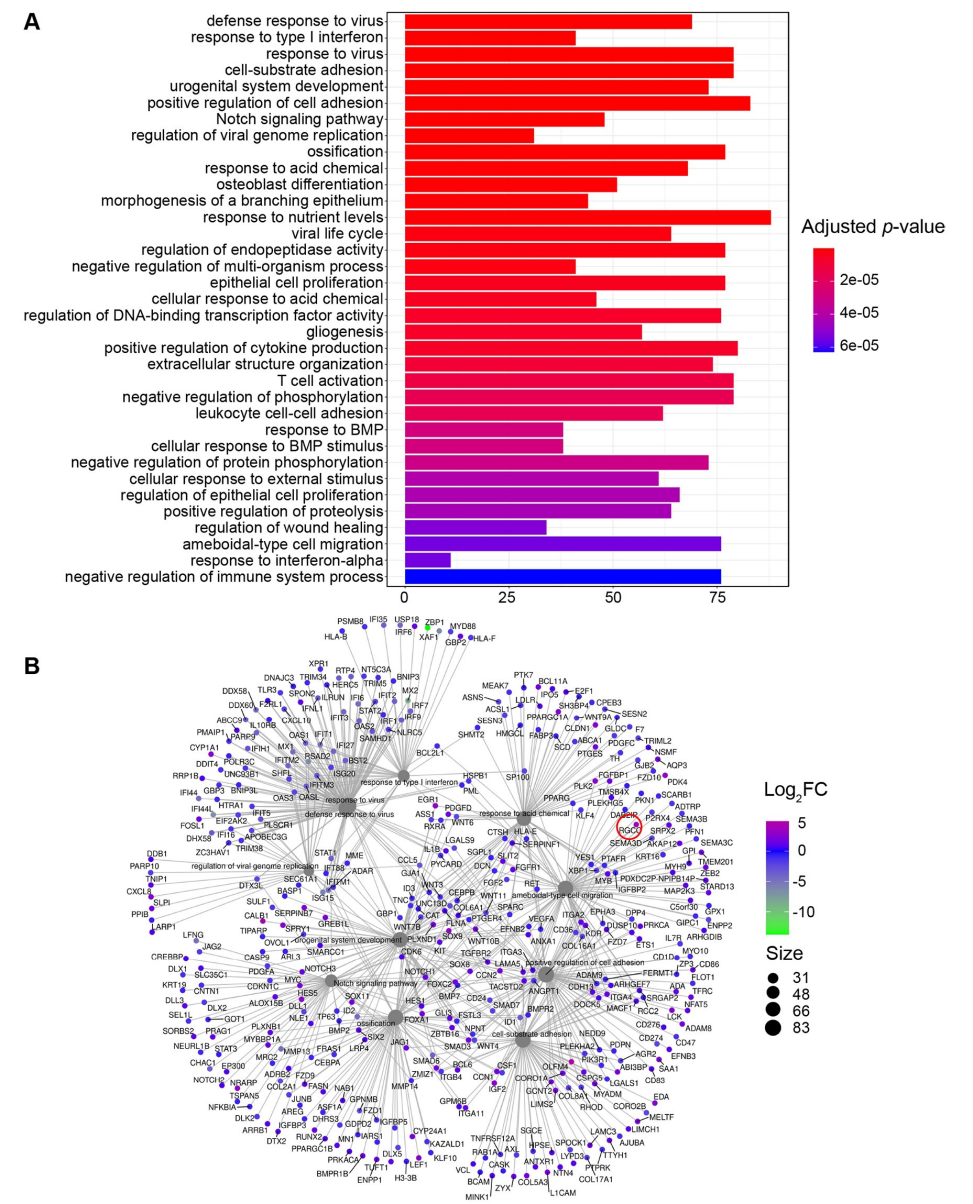
Transcriptomic responses of TIGKs to challenge with *P*. *gingivalis*. A) GO term classifications of pathways containing significant numbers (as denoted below figure) of differentially regulated genes in TIGK cells challenged with *P*. *gingivalis*. B) String analysis of differentially regulated genes in the GO term of ameboidal-type cell migration. *RGCC* is highlighted with a red circle.

To gain insight into pathways and effector molecules targeted by Ltp1, we interrogated our RNASeq database (GEO, # GSE159868) for TIGK genes related to proliferation and migration, and which were differentially expressed by infection with *P*. *gingivalis*. In the *P*. *gingivalis* challenge condition, the pathways: epithelial proliferation (GO:0050673), regulation of epithelial cell proliferation (GO:0050678) and ameboidal-type cell migration (GO:0001667), were enriched with 79, 68 and 77 differentially expressed genes respectively (Fig 3A). *RGCC* with a Log2FC of 4.19 was one of the genes most significantly induced by *P*. *gingivalis* infection in the GO term of ameboidal-type cell migration (Fig 3B).

**Fig 4:**
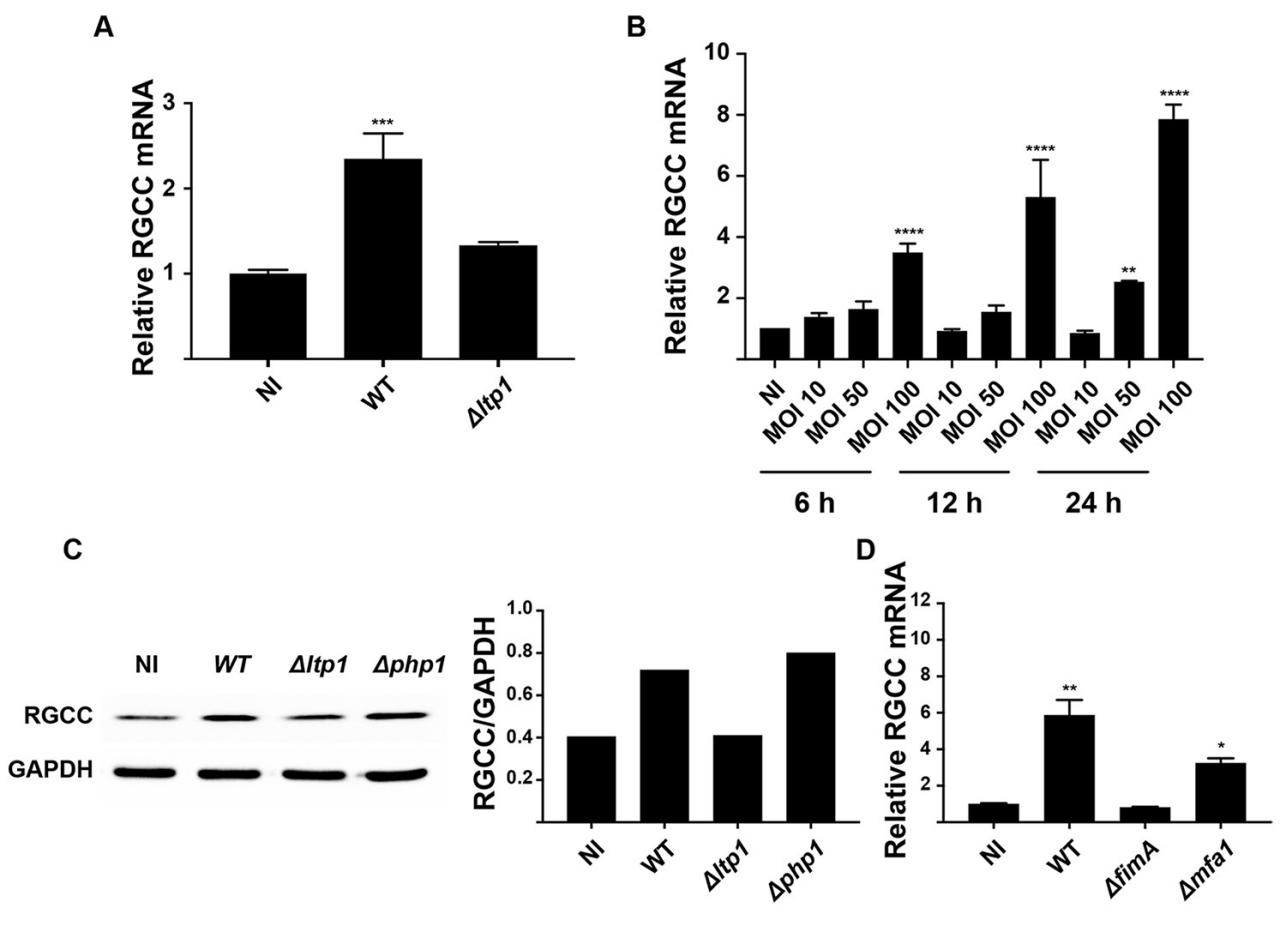
RGCC upregulation by *P*. *gingivalis* is Ltp-1 dependent. A) TIGK cells were challenged with *P*. *gingivalis* WT or Δ*ltp1* (MOI 100, 6h). Expression of RGCC mRNA was measured by qRT-PCR. Data are expressed relative to noninfected (NI) controls. B) TIGK cells were challenged with *P*. *gingivalis* WT at the times an MOI indicated. Expression of RGCC mRNA was measured by qRT-PCR. Data are expressed relative to noninfected (NI) controls. C) Left panel. Western blots of whole-cell lysates of TIGKs challenged with *P*. *gingivalis* WT, Δ*ltp1*, or Δ*php1* (MOI 100, 6h), probed with antibodies to RGCC or GAPDH as a loading control. Densiometric analysis of blots with ImageJ is shown in the right panel. D) TIGK cells were challenged with *P*. *gingivalis* WT, Δ*fimA*, or Δ*mfa1* (MOI 100, 6h). Expression of RGCC mRNA was measured by qRT-PCR. Data are expressed relative to noninfected (NI) controls. * p< 0.05, ** p < 0.01, **** p < 0.001 compared to NI.

Differential regulation of RGCC mRNA by *P*. *gingivalis*, and the requirement for Ltp1, was corroborated by qRT-PCR (Fig 4A). Additionally, RGCC mRNA amounts were increased in a time- and dose-dependent manner by *P*. *gingivalis* (Fig 4B), and immunoblotting showed that RGCC was increased at the protein level by infection with Ltp1-producing *P*. *gingivalis* (Fig 4C). A mutant of *P*. *gingivalis* lacking the major fimbrial structural protein (FimA) was unable to increase RGCC mRNA levels, whereas transcription of *RGCC* was upregulated by a mutant deficient in production of the minor fimbrial subunit protein Mfa1 (Fig 4D). FimA is an epithelial adhesin and invasin, and the Δ*fimA* mutant, but not the Δ*mfa1* mutant, is deficient in invasion into epithelial cells [30,31]. Hence, these data indicate the Ltp1 has to be produced intracellularly to regulate *RGCC* levels.

**Fig 5:**
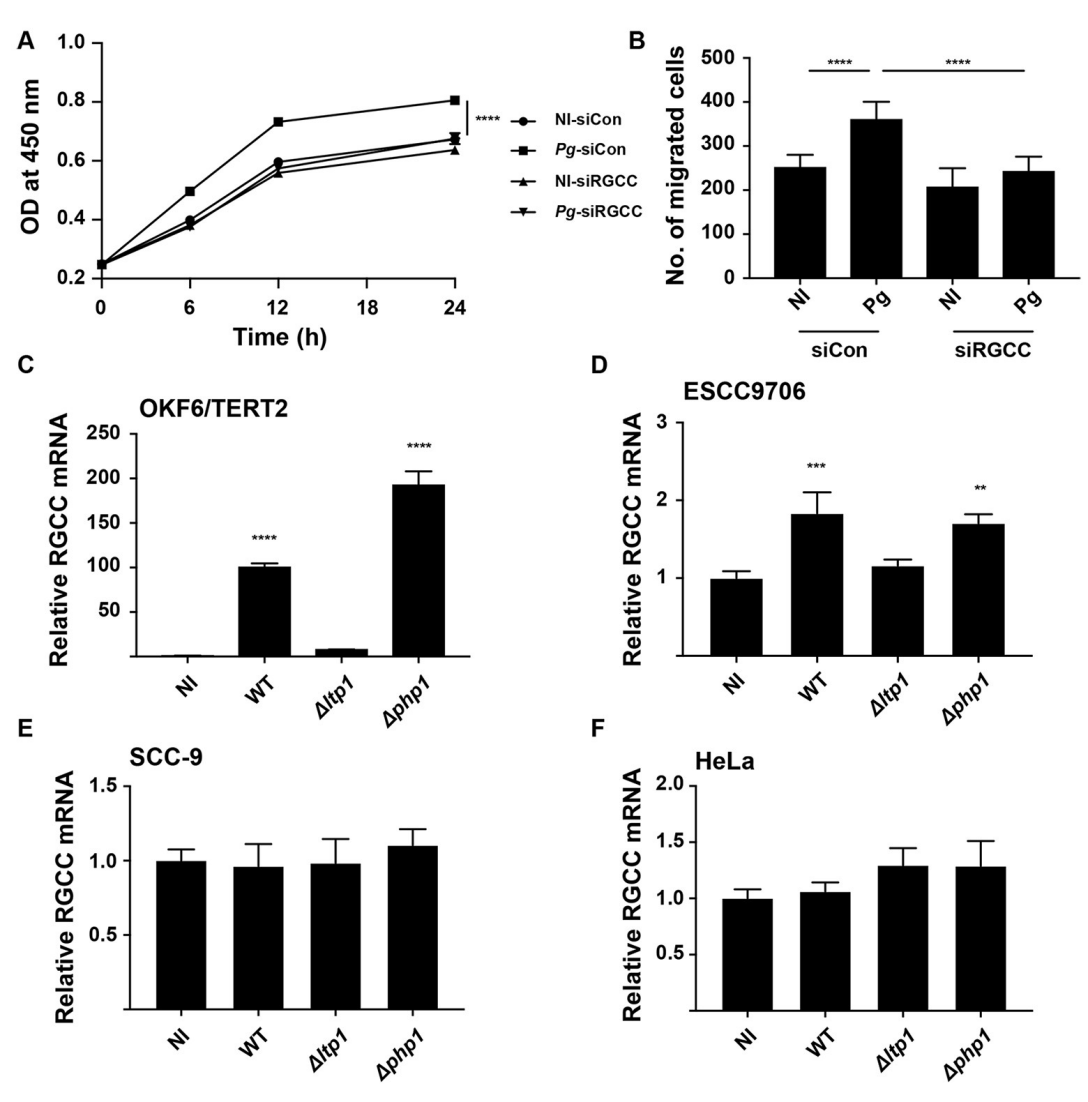
RGCC controls TIGK proliferation and migration in response to *P*. *gingivalis* and is regulated in different cell types. TIGK cells were transiently transfected with siRNA to RGCC (siRGCC) or scrambled siRNA (siCon) and challenged with *P*. *gingivalis* (Pg) or left uninfected (NI). A) Absorbance at 450 nm measured over the times indicated in a BrdU proliferation ELISA. B) Quantitative analysis of TIGK migration through matrigel-coated transwells. C-F) Cells as indicated were challenged with *P*. *gingivalis* WT, Δ*ltp1*, or Δ*php1*. Expression of RGCC mRNA was measured by qRT-PCR. Data are expressed relative to noninfected (NI) controls. ** p< 0.01, *** p < 0.005, **** p < 0.001.

To corroborate the involvement of RGCC in proliferation and migration of TIGKs, expression was reduced by siRNA. Both proliferation and migration of TIGKs in response to *P*. *gingivalis* infection were diminished in the *RGCC* knockdown condition (Fig 5A and 5B). Cell specificity of RGCC regulation was assessed using epithelial cells derived from different anatomical sites. *RGCC* mRNA was upregulated in an Ltp1-dependent manner in the OKF6 line, which are telomerase immortalized buccal mucosa cells (Fig 5C). Moreover, the high level of RGCC induction in OKF6 cells would indicate that the buccal mucosa is the more responsive to *P*. *gingivalis* in terms of RGCC activity than is the gingival epithelium. Esophageal squamous cell carcinoma cells (ESCC9706) also displayed a modest increase in *RGCC* mRNA expression (Fig 5D); however, *RGCC* regulation did not occur in tongue squamous carcinoma cells (SCC9) or in HeLa cells (Fig 5E and 5F).

### RGCC is regulated by the Akt-PTEN pathway

**Fig 6:**
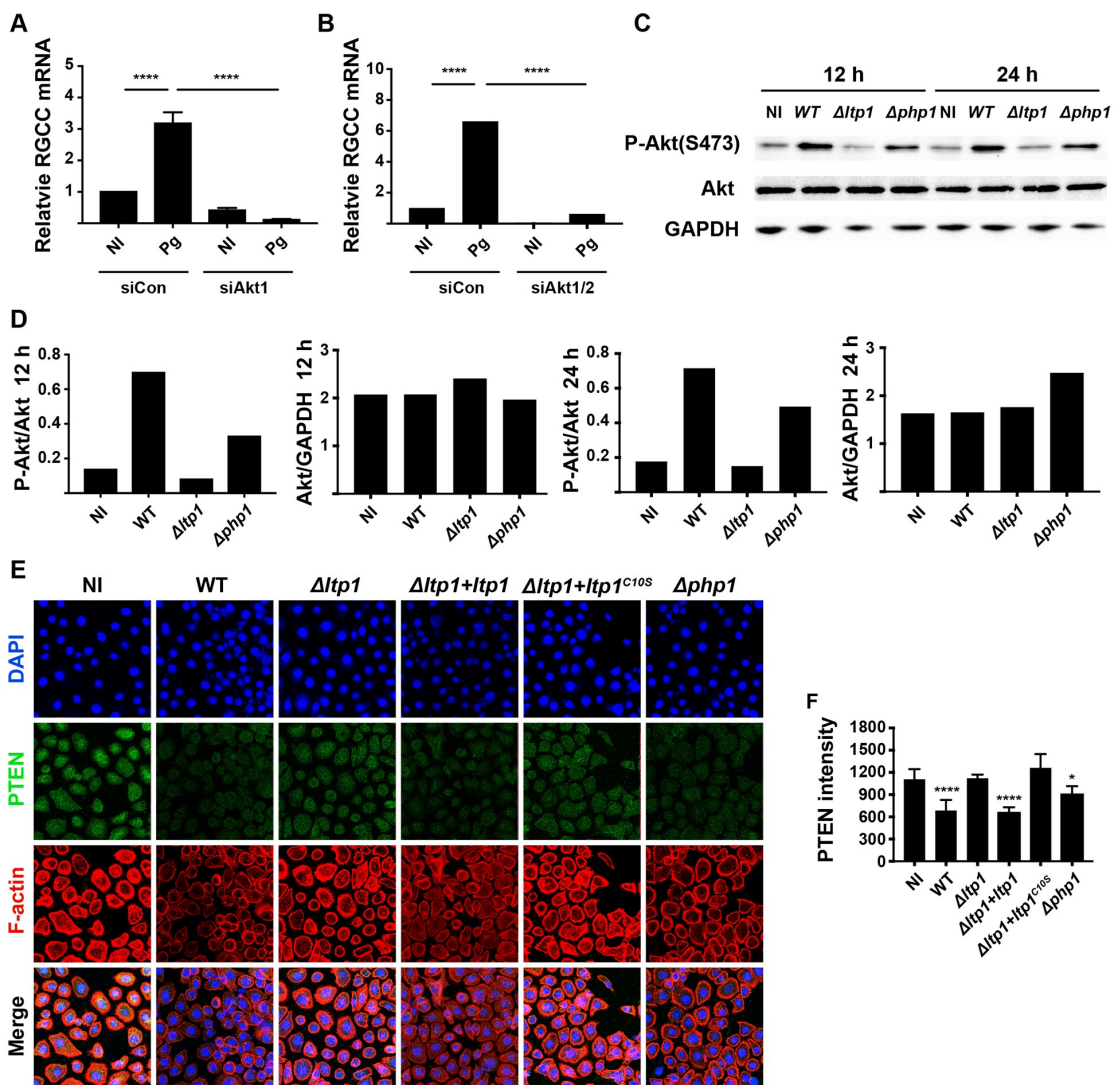
Control of RGCC by *P*. *gingivalis* involves Akt and PTEN. TIGK cells were transiently transfected with siRNA to Akt1 (A) or Akt1/2 (B) or scrambled siRNA (siCon) and challenged with *P*. *gingivalis* (Pg). Expression of RGCC mRNA was measured by qRT-PCR. Data are expressed relative to noninfected (NI) controls. C) Western blots of TIGKs challenged *P*. *gingivalis* WT, Δ*ltp1*, Δ*php1*, or left uninfected (NI) for 12 h or 24 h. Blots were probed with antibodies to Akt, phospho(P-) Akt (serine 473) or GAPDH as a loading control. D) Densiometric analysis of blot in (C) using ImageJ. E) Fluorescent confocal microscopy of TIGK cells challenged with *P*. *gingivalis* WT, Δ*ltp1*, Δ*php1*, Δ*ltp1*+*ltp1*, Δ*ltp1*+*ltp1*^C10S^, or left uninfected (NI). Cells were probed with PTEN antibodies and Alexa Fluor 488 secondary antibody (green). Actin (red) was stained with Texas Red-phalloidin, and nuclei (blue) stained with DAPI. Cells were imaged at magnification ×63, and shown are projections of z-stacks generated with Volocity. F) PTEN staining intensity was quantified in over 200 cells and normalized to the number of cells with Volocity software. * p< 0.05, **** p < 0.001 compared to NI.

In order to functionally dissect the mechanism by which Ltp1 impinges upon RGCC regulation, a phospho-antibody array was used to detect changes in phosphoproteins in TIGKs ectopically expressing Ltp1. Table 1 shows differentially regulated candidates (0.8 > FC > 1.2), of which increased phosphorylation of the PI3K-kinase p85-subunit alpha/gamma had most relevance to RGCC signaling. PI3K p85 phosphorylation links the Class I PI3K pathway to Akt [32] which controls RGCC [33]. The role of Akt was verified by siRNA which showed that knockdown of Akt1/2 or Akt1 alone prevented an increase in *P*. *gingivalis*-dependent RGCC transcription (Fig 6A and 6B). The phosphorylation/activation status of Akt following *P*. *gingivalis* infection was examined by immunoblotting. Fig 6C and 6D shows that *P*. *gingivalis* WT and Δ*php1* strains induce an increase in the phosphorylation of Atk at S473, which did not occur with the Δ*ltp1* strain, at both 12 and 24 h of infection. These results indicate that Ltp1 does not act on Akt directly. The PI3K/AKT signaling pathway can be opposed by PTEN, which dephosphorylates PIP3 to generate PIP2, thus blocking PI3K signaling [34]. Confocal microscopy revealed that PTEN levels in TIGKs were reduced by *P*. *gingivalis* strains producing functional Ltp1 (WT, Δ*ltp1*+*ltp1*, Δ*php1*, Fig 6E and 6F). However, the Ltp1 deficient mutant of *P*. *gingivalis* was unable to reduce PTEN levels and complementation with the catalytically dead Ltp1 C10S did not restore the parental phenotype. Thus, Ltp1 can decrease the amount of PTEN in TIGK cells in a phosphatase dependent manner, consistent with an increase in Akt1 activation.

**Table 1:** Changes in phosphoprotein expression in TIGK cells in response to ectopic expression of Ltp1.

Phosphoprotein	Signal ratios		Ratio change	p*-*value
	Control	Ltp1	Ltp1/control	
CaMK2-beta/gamma/delta (Phospho-Thr287)	0.30	0.43	1.44	<0.0001
Rho/Rac guanine nucleotide exchange factor 2 (Phospho-Ser885)	0.64	0.85	1.31	<0.0001
PI3-kinase p85-subunit alpha/gamma (Phospho-Tyr467/Tyr199)	0.56	0.67	1.20	<0.0001
PLC beta-3 (Phospho-Ser537)	1.50	1.15	0.77	0.0012
MEK1 (Phospho-Ser221)	1.37	1.04	0.76	<0.0001
Cortactin (Phospho-Tyr421)	1.99	1.51	0.76	<0.0001
MEK1 (Phospho-Thr291)	0.58	0.44	0.75	<0.0001
ERK1-p44/42 MAP Kinase (Phospho-Tyr204)	1.51	1.13	0.75	<0.0001
Ezrin (Phospho-Thr566)	0.35	0.26	0.72	0.002
VASP (Phospho-Ser157)	0.85	0.60	0.70	<0.0001
MEK1 (Phospho-Ser298)	2.02	1.38	0.68	<0.0001
Src (Phospho-Tyr418)	0.22	0.10	0.46	<0.0001

**Fig 7:**
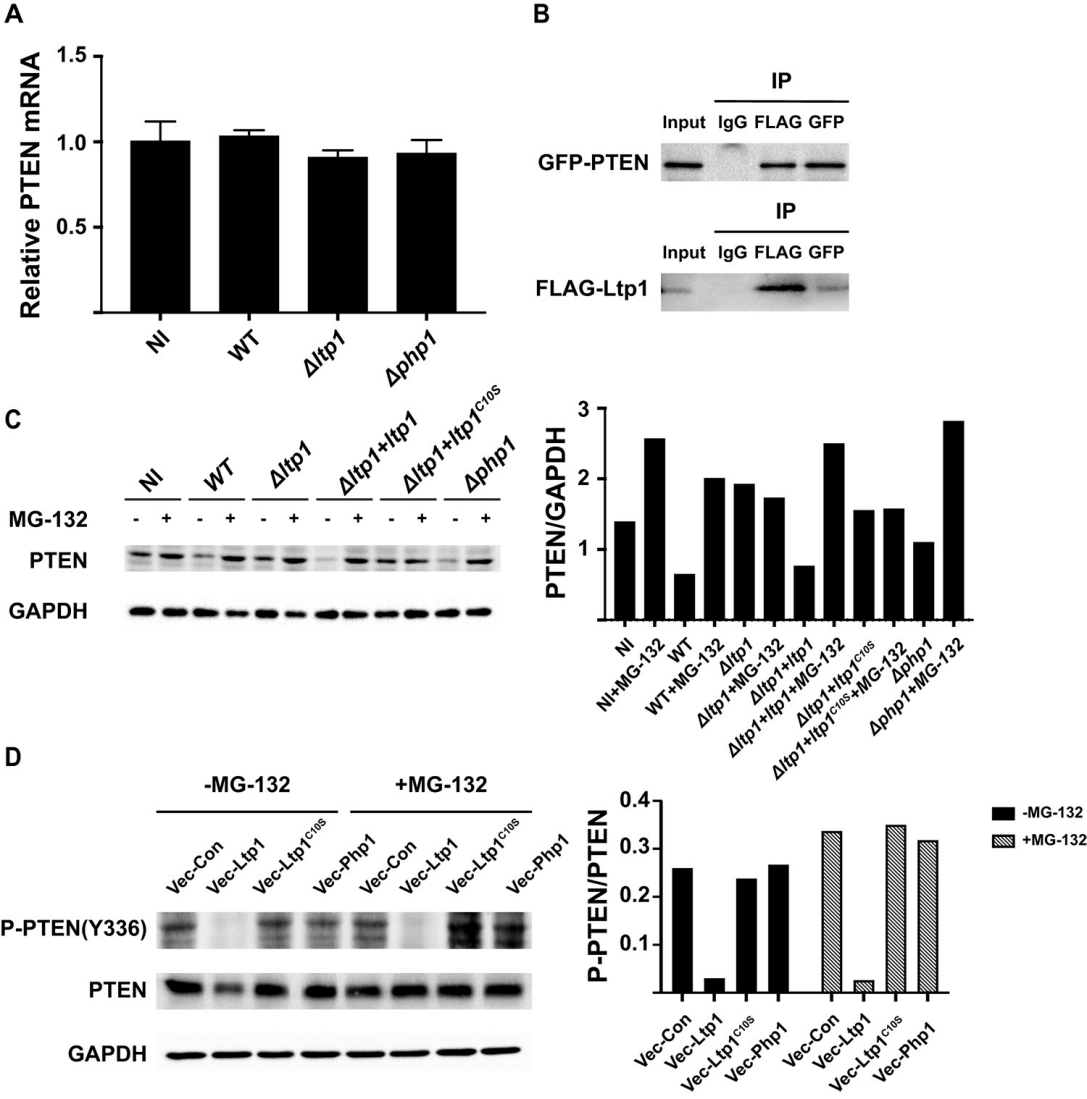
Ltp1 induces PTEN proteasomal degradation. A) TIGK cells were challenged with *P*. *gingivalis* WT, Δ*ltp1*, or Δ*php1*. Expression of PTEN mRNA was measured by qRT-PCR. Data are expressed relative to noninfected (NI) controls. B) TIGK cells were transiently transfected with GFP-PTEN and FLAG-Ltp1, and cross-linked with dithiobis(succinimidyl propionate). Lysates were immunoprecipitated with GFP, Ltp1 or control IgG antibodies. Immunoblots show cell lysates before immunoprecipitation (input) and immunoprecipitates (IP) probed with antibodies indicated. C. Left panel. Western blots of TIGKs challenged *P*. *gingivalis* WT, Δ*ltp1*, Δ*ltp1*+*ltp1*, Δ*ltp1*+*ltp1*^C10S^, Δ*php1*, or left uninfected (NI) with or without MG-132. Blots were probed with antibodies to PTEN, or GAPDH as a loading control. Right panel. Densiometric analysis of blot using ImageJ. D) Left panel. Western blots of TIGKs transiently transfected with Ltp1 (Vec-Ltp1), Php1 (Vec-Php1) or empty vector control (Vec-Con), with or without MG-132. Blots were probed with antibodies to phospho(P-) PTEN (tyrosine 336), PTEN, or GAPDH as a loading control. Right panel. Densiometric analysis of blot using ImageJ.

To investigate the nature of PTEN regulation by Ltp1, we first assayed PTEN mRNA levels. qRT-PCR demonstrated that Ltp1 had no effect on *PTEN* transcriptional activity (Fig 7A), suggesting post-transcriptional control. We next used co-precipitation to investigate the ability of Ltp1 to bind to PTEN. GFP-PTEN and FLAG-Ltp1 were ectopically expressed in TIGK cells and co-precipitated with antibodies to GFP or FLAG. As shown in Fig 7B, probing blots of immunoprecipitates with the complementary antibody established direct interactions between Ltp1 and PTEN. PTEN levels in the cell are regulated by phosphorylation at Y336, which protects the protein from ubiquitin-mediated degradation [35]. Thus, we reasoned that Ltp1 may affect the stability of PTEN, and immunoblotting verified that PTEN levels were diminished in TIGKs infected with *P*. *gingivalis* strains producing functional Ltp1 (Fig 7C). Moreover, regulation of PTEN levels by Ltp1 was lost when the proteasome inhibitor MG-132 was present. To determine the phosphorylation status of PTEN Y336 in response to LTP1, we ectopically expressed *ltp1*, *ltp1*C10S or *php1* in TIGK cells. Immunoblotting showed that catalytically active Ltp1, but neither inactive Ltp1C10S nor Php1, reduced phosphorylation of PTEN on Y336 (Fig 7D). Consistent with this, the amount of total PTEN protein was reduced by Ltp1, while stability of PTEN was enhanced in the presence of MG-132.

### RGCC controls Zeb2 induction by *P*. *gingivalis*

**Fig 8:**
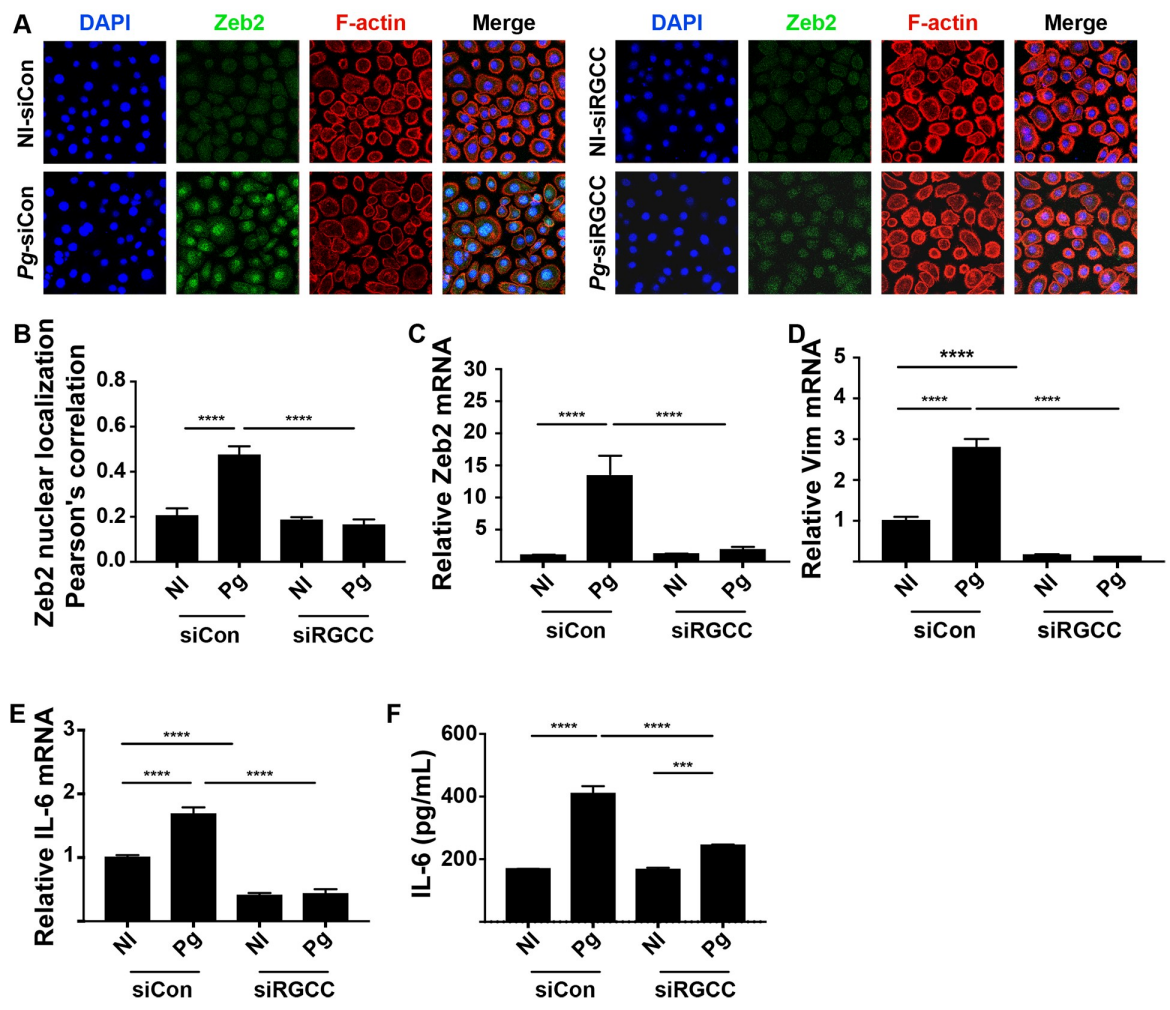
*P*. *gingivalis*-induced RGCC regulates Zeb2, IL-6 and in vivo pathogenicity. A) Fluorescent confocal microscopy of TIGK cells transiently transfected with siRNA to RGCC (siRGCC) or scrambled siRNA (siCon), and challenged with *P*. *gingivalis* (Pg) or left uninfected (NI). Cells were probed with Zeb2 antibodies as indicated, and Alexa Fluor 488 secondary antibody (green). Actin (red) was stained with Texas Red-phalloidin, and nuclei (blue) stained with DAPI. Cells were imaged at magnification ×63, and shown are projections of z-stacks generated with Volocity. B) Colocalization of Zeb2 with the nuclear region from the images in (A) using Pearson’s correlation in Volocity. C-E) TIGK cells were transiently transfected with siRNA to RGCC (siRGCC) or scrambled siRNA (siCon) and challenged with *P*. *gingivalis* (Pg). Expression of mRNA for Zeb2 (C), vimentin (D), or IL-6 (E) was measured by qRT-PCR. Data are expressed relative to noninfected (NI) controls. F) ELISA of IL-6 protein in TIGK supernatants. *** p < 0.005, **** p < 0.001.

**Fig 9:**
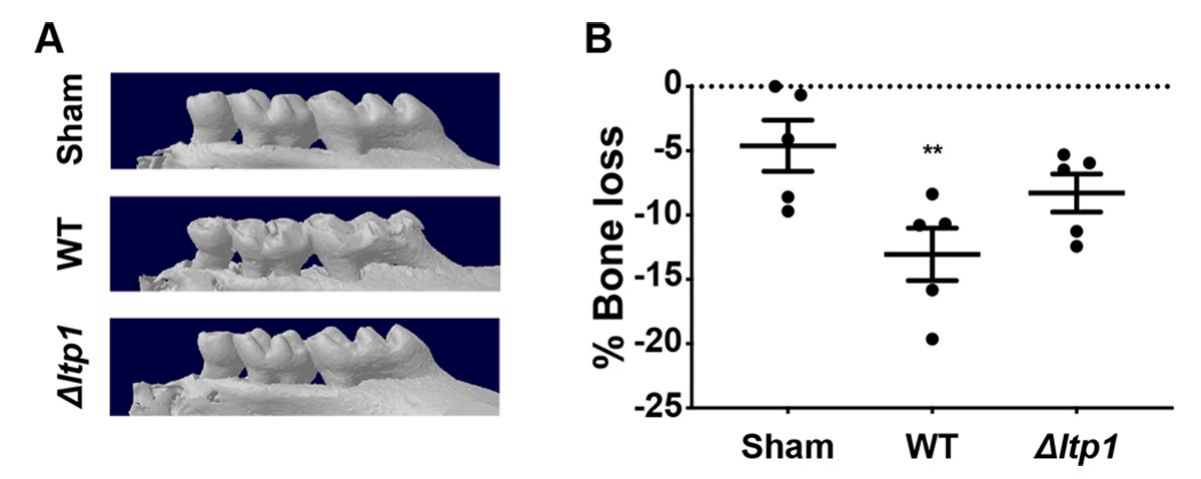
Alveolar bone loss in mice following infection with *P*. *gingivalis* WT or Δ*ltp1* mutant was determined by μCT analysis. A) Representative images of the maxillary molars reconstructed with NRecon Reconstruction software. B) Reconstructed images were analyzed along the sagittal slice to determine the distance between the ABC (alveolar bone crest) and the CEJ (cementoenamel junction) relative to the root length of the tooth. The data are the averages and scatter for each mouse from 12 measurements across both the first and second molars relative to the sham-treated mice and are expressed as means ± SE. ** p < 0.01 by one-way analysis of variance relative to sham infection.

RGCC can facilitate EMT through Smad/Zeb2 signaling in colorectal cancer cells [36], and as *P*. *gingivalis* also transitions gingival epithelial cells toward EMT through Zeb2, we hypothesized that RGCC may also control Zeb2 responses to *P*. *gingivalis*. Confocal microscopy found that upregulation and nuclear localization of Zeb2 were attenuated in *P*. *gingivalis*-infected cells in which expression of *RGCC* was reduced by siRNA (Fig 8A and 8B). qRT-PCR established that lower levels of Zeb2 in siRGCC cells resulted from transcriptional downregulation (Fig 8C). Further, siRNA knockdown of RGCC also prevented *P*. *gingivalis*-dependent upregulation of mRNA for vimentin (Fig 8D), a mesenchymal marker controlled by Zeb2 [37]. In TIGKs, Zeb2 also controls production of IL-6 mRNA in response to *P*. *gingivalis* [17]. We found that both IL-6 transcriptional activity and protein secretion were reduced in response to *P*. *gingivalis* following siRNA knockdown of *RGCC* (Fig 8E and 8F). The proinflammatory cytokine IL-6 is correlated with the level of periodontal tissue destruction [38], and thus we tested the virulence of the Ltp1 deficient mutant. Fig 9 shows that in a murine model of alveolar bone loss, oral infection with the Δ*ltp1* mutant resulted in significantly reduced bone loss compared to the parental strain. Cumulatively, these data suggest that the Akt-RGCC pathway is one mechanism by which Zeb2 is controlled, and this pathway is involved in EMT and cytokine production in response to *P*. *gingivalis*, and can ultimately impact periodontal pathogenicity.

## Discussion

Tyrosine phosphorylation of bacterial proteins has emerged as a key regulatory modification contributing to stress responses, cell division, DNA metabolism, cell wall polysaccharide biosynthesis and community development [24,25,39]. Tyrosine phosphorylation is tightly controlled by the reciprocating action of bacterial tyrosine (BY) kinases and tyrosine phosphatases (PTPs). *P*. *gingivalis* is unusual among Gram-negatives in that it possesses both a low molecular weight (LMW)-PTP and a polymerase/histidinol phosphatase (PHP), both of which contribute to community formation with the oral partner species *Streptococcus gordonii* [7]. In addition to its biofilm lifestyle, in the oral cavity *P*. *gingivalis* engages gingival epithelial cells in an intricate molecular dialogue which can disrupt tissue and immune homeostasis. Indeed, *P*. *gingivalis* can suppress apoptotic cell death, accelerate cell cycle progression, enhance cell migration, and induce a partial EMT in gingival epithelial cells [2,4,40–42]. A wealth of information is available regarding specific host effector molecules subverted by *P*. *gingivalis*; however, the effector molecules and signaling pathways leading to these adaptations are still poorly understood.

Numerous host-bacteria interactions depend on secreted bacterial phosphatases which can function within host cells [39,43]. For example, the *Staphylococcus aureus* LMW-PTP, PtpA, is secreted during macrophage infection and interacts with the cytoskeletal associated protein, coronin-1A [44]. *Mycobacterium tuberculosis* secretes two LMW-PTPs, PtpA and PtpB, into the macrophage cytosol where they disrupt key components of the endocytic pathway and cause an arrest of phagosome maturation [45,46]. Both *Yersinia* and *Salmonella* utilize the type III secretion system to deliver tyrosine phosphatases into epithelial cells where they uncouple multiple signal transduction pathways [47,48]. In this study, we show that the LMW-PTP Ltp1, but not the PHP, Php1, can be secreted by *P*. *gingivalis* both into the extracellular milieu and within gingival epithelial cells. *P*. *gingivalis* has a high capacity for internalization within epithelial cells [49], and while the organism can traffic to autophagosomes and early endosomes [50,51], a population of intracellular organisms remains in the cytoplasm [52]. We found that within epithelial cells, Ltp1 from *P*. *gingivalis* co-localized with the nuclear region. While there are a number of pathways by which bacterial proteins can translocate to the nucleus [53], tyrosine phosphorylation/dephosphorylation is involved in the shuttling of proteins into and out of the nucleus. For example, tyrosine phosphorylation of the Hippo pathway effector Yes-associated protein (YAP) limits interaction with exportin proteins and increases nuclear retention [54]. Interaction of YAP1 with the tyrosine phosphatase SHP2 then promotes accumulation of SHP2 in the nucleus [55]. As PTEN can translocate to the nucleus [56], its interaction with Ltp1 may effectuate Ltp1 nuclear localization.

In *P*. *gingivalis*, proteins are secreted by the type IX secretion system (T9SS) [57]. Ltp1 lacks the consensus C-terminal domain required for recognition by the T9SS, hence secretion of Ltp1 would suggest either non-canonical recognition by the T9SS, or the existence of an alternative secretion system in *P*. *gingivalis*. Alternatively, Ltp1 may be packaged in outer membrane vesicles (OMVs), although proteomic studies have yet to identify Ltp1 in these structures [58,59]. Moreover, the loss of Ltp1 function demonstrated by an invasion defective mutant of *P*. *gingivalis* (Δ*fimA*) would indicate that Ltp1 is being secreted inside the epithelial cells by intracellular bacteria.

Ltp1 was required for *P*. *gingivalis* to enhance epithelial cell proliferation and migration. Interrogation of our RNA-Seq database, along with corroboration by siRNA suppression, identified RGCC as involved in this property of *P*. *gingivalis*. RGCC regulates cell cycle progression along with cell differentiation and fibrosis [33]. RGCC is highly expressed in a number of tumors [60] and can promote tumor cell proliferation and EMT [61], although the functionality of RGCC can be cell and context specific [33]. RGCC binding partners and substrates include cyclin-dependent kinase 1 (CDK1), polo-like kinase 1 (PLK1) and Akt, and RGCC is centrosome-associated during mitosis [60,62,63]. In addition, RGCC can induce EMT and enhance the migration and invasion of lung adenocarcinoma cells through increased MMP2 and MMP9 production and activity [64,65]. Induction of EMT by RGCC in A549 lung adenocarcinoma cells occurs through the activation of NF-κB signaling pathway [65], whereas in colon adenocarcinoma cells, RGCC activates the Smad/Zeb2 pathway [36].

**Fig 10:**
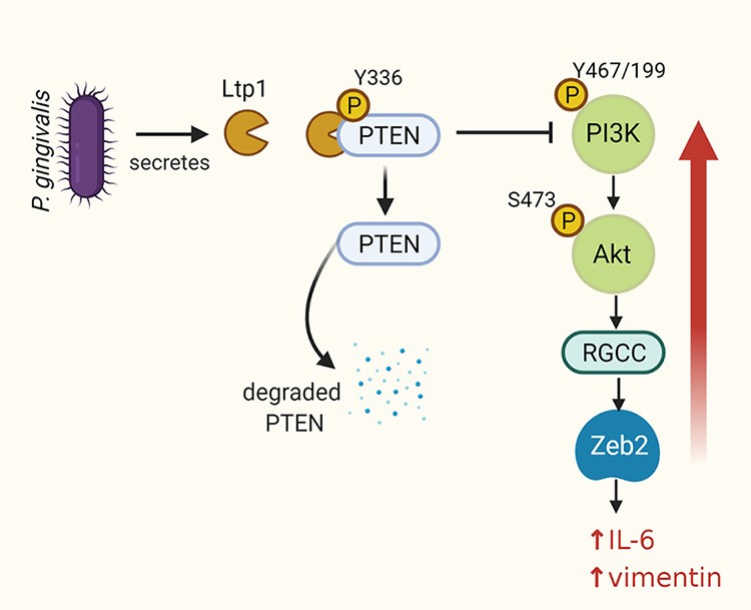
Schematic (not to scale) of the impact of Ltp1 secreted within epithelial cells by *P*. *gingivalis*. Ltp1 dephosphorylates PTEN leading to proteasomal degradation. A reduction of PTEN levels relieves suppression of PI3K/Akt which upregulates RGCC and Zeb2.

Our data suggest that RGCC occupies a signaling hub in the manipulation of the host cell by *P*. *gingivalis* through the Ltp1 phosphatase (shown schematically in Fig 10). A phosphoprotein antibody array analysis of TIGK cells expressing ectopic Ltp1 indicated an increase in the activity of PI3K which is upstream of Akt, a regulator of RGCC [33,66]. Immunoblotting showed that in TIGKs infected with a Ltp1-deficient mutant there was a decrease in S473 phosphorylation, which is required for full Akt activity, and siRNA suppression of AKT prevented an increase in RGCC transcription in response to *P*. *gingivalis*. Hence PI3K/Akt signaling appears to be a target of Ltp1, but acting through an inhibitor to cause increased phosphorylation. The phosphatidylinositol-3,4,5-trisphosphate 3-phosphatase (PTEN) preferentially dephosphorylates phosphoinositide substrates and thus inhibits PI3K action by negatively regulating intracellular levels of phosphatidylinositol-3,4,5-trisphosphate [67]. Several lines of evidence indicated that dephosphorylation of PTEN by Ltp1 increases proteasomal degradation, thus relieving suppression of PI3K/Akt. In TIGK cells infected with functional Ltp1-producing *P*. *gingivalis*, PTEN was reduced at the protein, but not mRNA, level. PTEN co-precipitated with Ltp1 ectopically expressed in TIGKs, and Ltp1 expression also decreased phosphorylation of PTEN on Y336, a posttranslational modification which inhibits ubiquitin-mediated degradation. Finally, chemical inhibition of proteasomal activity reduced degradation, but not dephosphorylation, of PTEN by Ltp1. Hence, PTEN represents a novel target for an intracellular LMW-PTP, and provides an access point for disruption of cell signaling.

*P*. *gingivalis* initiates a partial EMT in gingival epithelial cells through regulation of the transcription factors Zeb1, Zeb2, and SNAI1/2 [17,40,68]. Differential regulation of Zeb2 by *P*. *gingivalis* has been shown to be mediated through pathways involving β-catenin and FOXO1 [17]. Zeb2 is also downstream of RGCC [36], and we show here that regulation of RGCC by *P*. *gingivalis* also controls Zeb2 and the associated expression of mesenchymal markers such as vimentin. Multi-tiered control of Zeb2 by *P*. *gingivalis* may have relevance to the oral environment, in which *P*. *gingivalis* resides in a complex heterotypic community [2,4]. Some partner species of *P*. *gingivalis*, such as *S. gordonii*, can antagonize the FOXO1-Zeb2 axis [17]. Hence, a redundancy in Zeb2 activation mechanisms may allow *P*. *gingivalis* to maintain tonic levels of Zeb2 even in the presence of antagonistic species. Zeb2 can also control the expression of inflammatory cytokines, and we found that RGCC suppression reduced the level of IL-6 secretion in response to *P*. *gingivalis*. IL-6 can incite bone resorption and MMP production [69], and is a potential diagnostic marker for periodontal destruction [70]. Activation of RGCC by Ltp1 may thus contribute to the immune dysbiosis elicited by *P*. *gingivalis* at the gingival interface. The inability of the Ltp1-deficient mutant of *P*. *gingivalis* to cause alveolar bone loss in a murine model of periodontal disease established the importance of this process in vivo.

While RGCC may have pleiotropic functions in different settings, depending on cell lineage, activation mechanisms and protein levels, a number of cancers are associated with upregulation of *RGCC* mRNA including colon, ovarian, breast and prostate [71]. In colorectal cancer (CRC) tissues, RGCC expression was significantly correlated with invasive and aggressive characteristics of tumor cells, as well as poor survival of the patients. Additionally, RGCC overexpression promoted proliferation, migration and tumorigenic growth of human CRC cells *in vitro* and *in vivo* [36]. PTEN is a well-characterized tumor suppressor that is mutated or lost in a large number of carcinomas. Of relevance to the oral environment, PTEN deficiencies have been shown to contribute to the development and progression of head and neck squamous cell carcinomas [72]. Ltp1-mediated reduction of PTEN and upregulation of RGCC could, therefore, represent a potential mechanistic basis underlying the emerging association between *P*. *gingivalis* and OSCC [10,12,73].

## Materials and methods

### Ethics statement

Animal experiments were performed under University of Louisville IACUC approved protocol 18237.

### Bacteria and cell culture

*P*. *gingivalis* strains were cultured anaerobically at 37°C in Trypticase soy broth (TSB) supplemented with 1 mg/ml yeast extract, 5 μg/mL hemin, and 1 μg/mL menadione. When necessary, erythromycin (10 μg/mL) or tetracycline (1 μg/mL) was added to the medium for isogenic mutants. *Escherichia coli* was grown aerobically at 37°C with shaking in Luria-Bertani broth containing ampicillin (100 μg/ml) or kanamycin (50 μg/mL) when required. All bacterial strains are listed in S1 Table.

Human telomerase immortalized gingival keratinocytes (TIGK) and OKF6/TERT cells were maintained in Dermalife-K serum free culture medium (Lifeline Cell Technology, Carlsbad, CA). EC9706, SCC9 and HeLa cells were cultured in DMEM supplemented with 10% fetal bovine serum (FBS). Cells were grown to about 80% confluence were challenged with bacteria at a multiplicity of infection (MOI) of 100 for 6 h, unless otherwise noted. Detailed information for eukaryotic cells is listed in S2 Table.

### Antibodies and reagents

Rabbit polyclonal antisera to recombinant Ltp1 and Php1 were generated by Abgent (San Diego, CA). PTEN, AKT, p-AKT(S473), GAPDH, and GFP antibodies were from Cell Signaling Technology (Danvers, MA). α-tubulin and PARP antibodies and fluorescent F-actin were from ThermoFisher (Waltham, MA). p-PTEN(Y336) antibody was from Abcepta (San Diego, CA). Zeb2 antibody was from Abcam. FLAG M2 antibody was from Sigma. RGCC antibody was from Novus Biologicals (Littleton, CO). MG-132 (carbobenzoxy-L-leucyl-L-leucyl-L-leucine) was from EMD Biosciences (Rockland, MA) and was used at 10 μM.

### Cell fractionation

TIGK cells were harvested with trypsin-EDTA, and cytoplasmic and nuclear proteins were separated with NE-PER Nuclear and Cytoplasmic Extraction reagent (ThermoFisher) according to manufacturer’s instructions. Nuclear lysates were concentrated using Amicon Ultra concentrators (Millipore, Burlington, MA) prior to immunoblotting.

### Plasmid preparation, RNA interference and transfections

*ltp1* and *php1* were amplified by PCR (primers in S3 Table) and cloned into pCDNA3.1(+) (Invitrogen, Carlsbad, CA). A C10S mutation of *ltp1* was generated by a PCR-based site-directed mutagenesis kit (New England Biolabs, Ipswich, MA) using the primers in S3 Table. GFP-tagged PTEN was purchased from Addgene [74]. All constructs were confirmed by sequencing. Knockin transfection of TIGK cells was for 36 h, and siRNA knockdown was for 48 h using Lipojet transfection agent (SignaGen). All knockdowns were confirmed by *q*PCR.

### Quantitative reverse transcription PCR

Total mRNA was isolated using an RNAeasy plus kit (Qiagen, Germantown, PA) and reverse transcribed into cDNA using a high capacity cDNA reverse transcription kit (2 μg per reaction, Applied Biosystems, Waltham, MA) Quantitative PCR analysis were performed with the Biosystems TaqMan fast universal master mix and TaqMan gene expression assays (Thermo Fisher) on an Applied Biosystems QuantStudio 3. Relative mRNA expression was evaluated using the 2^-ΔΔCT^ method [17], with GAPDH internal control.

### Immunofluorescence and confocal laser scanning microscopy

TIGK cells were grown on chamber slides, challenged with bacteria, washed three times in PBS, and fixed with 4% paraformaldehyde for 10 min. After permeabilization with 0.2% Triton X-100 for 10 min at room temperature (RT), cells were blocked for 45 min in 5% bovine serum albumin (BSA). Cells were reacted with primary antibodies, followed by Alexa Fluor 488-labelled secondary antibodies, Texas Red-phalloidin and DAPI as previously described [17]. Slides were viewed by laser scanning confocal microscopy (SP8; Leica). Images were acquired and analyzed using Volocity 6.3 (PerkinElmer, Waltham, MA).

### Immunoblotting

Cells were lysed on ice in RIPA buffer containing Protease and PhosSTOP phosphatase inhibitor (Roche, San Francisco, CA). Lysates were separated by 12% SDS-PAGE, transferred to nitrocellulose membranes, and blocked with 5% BSA in Tris-buffered saline containing 0.1% Tween 20 (TBST). Membranes were incubated at 4°C overnight with primary antibodies, followed by 1 h incubation with HRP-conjugated secondary antibodies at RT. Immunoreactive bands were detected using an ECL kit (ThermoFisher) and a ChemiDoc XRS Plus (Bio-Rad, Hercules, CA).

### Preparation of *P*. *gingivalis* supernatants

*P*. *gingivalis* cultures (OD600 nm 1.0) were centrifuged at 14,000 x g for 10 min at 4°C. The supernatants were filtered (0.2 μm) and concentrated using Amicon Ultra concentrators.

### Immunoprecipitation

Cells were lysed in IP buffer (0.5% Nonidet P-40, 250 mM NaCl, 5 mM EDTA, and 50 mM Tris) containing Protease Inhibitor (Roche). Lysates were incubated with 1 μg of antibodies at 4°C overnight, followed by addition of 25 μL of protein G-conjugated agarose beads (Thermo Fisher). Immunoprecipitates were washed and separated using a magnetic separation rack, and eluted by boiling with SDS-PAGE sample buffer for immunoblotting.

### Enzyme-linked immunosorbent assay

The concentration of IL-6 in TIGK culture supernatants was determined with the Quantikine Human IL-6 (R&D Systems, Minneapolis, MN) according to manufacturer’s protocol.

### Phospho antibody array

TIGK cells were lysed in RIPA buffer and 40 μg were biotinylated and conjugated to a Phospho Antibody Array (Full Moon Biosystems, Sunnyvale, CA), which contains site-specific antibodies and phospho site-specific antibodies against 143 proteins related to cytoskeleton. Briefly, the cell lysates were incubated in labeling buffer with Biotin/dimethylformamide at RT for 2 h. Each array was blocked and incubated in a coupling chamber with labeled cell lysates at RT for 2 h. The arrays were washed and submerged in Cy3-Streptavidin solution at RT for 45 min in the dark, dried with compressed nitrogen, and scanned on an Axon GenePix Array Scanner (Axon Instruments, Inc, Union City, CA). Average signal intensity of replicate spots was normalized to the median signal. Using the normalized signal intensity of replicate spots for each pair of site-specific antibody and phospho site-specific antibody, the signal ratio of the paired antibodies was determined. The ratio change between treatment and control samples was calculated.

### Matrigel invasion, proliferation and apoptosis assays

Cell migration was measured using a BD BioCoat Matrigel Invasion Chamber (BD Biosciences, San Jose, CA) as described previously [17]. Proliferation of TIGKs was assessed using a BrdU Cell Proliferation ELISA kit (Abcam) according to the manufacturer’s protocol. Briefly, TIGKs were labeled with BrdU 2 h prior to the end of infection. Cells were fixed, and incubated with 100 μL/well anti-BrdU monoclonal antibody at RT for 1 h, followed by peroxidase goat anti-mouse IgG conjugate and its substrate. Spectrophotometric measurements were at 450 nm.

### Murine alveolar bone loss

BALB/cByJ mice, 10–12 weeks old, were obtained from Jackson Labs. Mice were fed a standard diet with water *ad libitum*. *P*. *gingivalis* 33277 or Δ*ltp1* (10^9^ cfu) were suspended in 0.1 ml of sterile PBS with 2% carboxymethylcellulose (CMC) and orally inoculated into mice at two-day intervals over a twelve-day period. A control group of mice were mock-inoculated with PBS and CMC alone. Forty-two days after the last infection, mice were euthanized and skulls were subjected to μCT scanning (SKYSCAN 1174, Bruker). Bone loss was assessed by measuring the distance between the alveolar bone crest and the cementoenamel junction at 14 interdental points between the first and second maxillary molars.

### Statistical analysis

Statistical analysis was performed using the ANOVA with Tukey’s multiple comparison test by GraphPad Prism V8. p values < 0.05 were considered significant. All data presented were performed with at least 3 biological replicates and two technical replicates and are expressed as mean ± SD unless otherwise noted.

## Supporting information

S1 Table Bacterial strains used in this study. (PDF)

S2 Table Eukaryotic cells used in this study. (PDF)

S3 Table Primers used in this study. (PDF)
