# Supplementary material for: A bacterial tyrosine phosphatase modulates cell proliferation through targeting RGCC
Source: PLoS Pathog. 2021 May 20;17(5):e1009598. doi: 10.1371/journal.ppat.1009598 (PMC8172045; doi:10.1371/journal.ppat.1009598)
Supplement: S1 Table — (PDF) [file ppat.1009598.s001.pdf]

Table S1. Bacterial Strains used in this study

Strain	Relevant characteristics	Source or reference
P. gingivalis		
33277	Type strain	ATCC
Δ*fimA*	Isogenic mutant of 33277 lacking major fimbrial gene *fimA*, Erm^r	[1]
Δ*mfa1*	Isogenic mutant of 33277 lacking minor fimbrial gene *mfa1*, Erm^r	[2]
Δl*tp1*	Isogenic mutant of 33277 lacking the low molecular tyrosine phosphatase gene *ltp1*, Erm^r	[3]
Δ*ltp1+ltp1*	Isogenic mutant of 33277 lacking the low molecular tyrosine phosphatase gene *ltp1*, and complemented with PT-COW-Ltp1, expressing functional Ltp1. Erm^r and Tet^r	[3]
Δ*ltp1+ltp1*^{C10S}	Isogenic mutant of 33277 lacking the low molecular tyrosine phosphatases gene *ltp1*, and complemented with PT-COW-Ltp1^{C10S}, expressing catalytically dead Ltp1. Erm^r and Tet^r	[3]
Δ*php1*	Isogenic mutant of 33277 lacking the tyrosine phosphatase gene *php1*, Erm^r	[4]
Escherichia coli		
TOP10	Chemically competent cells	Invitrogen

[a]Erm^r, Tet^r, and Amp^r indicate resistance to erythromycin, tetracycline, and ampicillin, respectively.

References

1. Yilmaz O, Watanabe K, Lamont RJ. Involvement of integrins in fimbriae-mediated binding and invasion by *Porphyromonas gingivalis.* Cell Microbiol 2002; 4: 305-314.
2. Park Y, Simionato MR, Sekiya K, Murakami Y, James D, Chen W*, et al.* Short fimbriae of *Porphyromonas gingivalis* and their role in coadhesion with *Streptococcus gordonii.* Infect Immun 2005; 73: 3983-3989.
3. Maeda K, Tribble GD, Tucker CM, Anaya C, Shizukuishi S, Lewis JP*, et al.* A P*orphyromonas gingivalis* tyrosine phosphatase is a multifunctional regulator of virulence attributes*.* Mol Microbiol 2008; 69: 1153-1164.
4. Jung YJ, Miller DP, Perpich JD, Fitzsimonds ZR, Shen D, Ohshima J*, et al. Porphyromonas gingivalis* tyrosine phosphatase Php1 promotes community development and pathogenicity*.* mBio 2019; 10: e02004-19.
