# Supplementary material for: A bacterial tyrosine phosphatase modulates cell proliferation through targeting RGCC
Source: PLoS Pathog. 2021 May 20;17(5):e1009598. doi: 10.1371/journal.ppat.1009598 (PMC8172045; doi:10.1371/journal.ppat.1009598)
Supplement: S2 Table — (PDF) [file ppat.1009598.s002.pdf]

Table S2. Eukaryotic cells

Cell name	Relevant characteristics	Reference or source
TIGK	Telomerase immortalized gingival epithelial cells	[1]
OKF6/TERT2	Telomerase immortalized oral mucosa cells	[2]
EC9706	Derived from esophageal squamous cell carcinoma	ATCC
SCC-9	Derived from tongue squamous cell carcinoma	ATCC
HeLa	Derived from cervical cancer	ATCC

References

1. Moffatt-Jauregui CE, Robinson B, de Moya AV, Brockman RD, Roman AV, Cash MN, *et al.* Establishment and characterization of a telomerase immortalized human gingival epithelial cell line. J Periodontal Res 2013; 48: 713-721.
2. Dickson MA, Hahn WC, Ino Y, Ronfard V, Wu JY, Weinberg RA, *et al.* Human keratinocytes that express hTERT and also bypass a p16^{INK4a}-enforced mechanism that limits life span become immortal yet retain normal growth and differentiation characteristics. Mol Cell Biol 2000; 20: 1436-1447.
