# Supplementary material for: A bacterial tyrosine phosphatase modulates cell proliferation through targeting RGCC
Source: PLoS Pathog. 2021 May 20;17(5):e1009598. doi: 10.1371/journal.ppat.1009598 (PMC8172045; doi:10.1371/journal.ppat.1009598)
Supplement: S3 Table — (PDF) [file ppat.1009598.s003.pdf]

Table S3. Primers used in this study

Name	Primer sequence (5' – 3')	Function
RTLtp1F	ACGGGAGAGCGAAGTGAGAT	Reverse-transcriptase PCR primers to determine *ltp1* expression
RTLtp1R	GTCTGCCGAGGCTGTCTTT	
RTPhp1F	GCGATCAAAAAGCAAGGCGT	Reverse-transcriptase PCR primers to determine *php1* expression
RTPhp1R	AGAAGCCTCGGACAGCAATC	
Ltp1F	CGGGATCCCGATGAAGCCACATAAAATCCTATTCGTATG	Generation of pcDNA3.1-Ltp1
Ltp1R	GGAATTCCTCAGTCGCATGCGGATTGG	
Php1F	CGGGATCCCATGTTTTCTATTTTCAAGCGAAAAAC	Generation of pcDNA3.1-Php1
Php1R	GGAATTCCCTAAGAAAACAATCGGGTGTTG	
FLtp1F	GCTCGGATCCATGAAGCCACATAAAATCCTATTCGTATG	Generation of pcDNA3.1-Flag-Ltp1
FLtp1R	AGTGGCGGCCGCTCAGTCGCATGCGGATTGG	
FPhp1F	GCTCGGATCCATGTTTTCTATTTTCAAGCGAAAAAC	Generation of pcDNA3.1-Flag-Php1
FPhp1R	AGTGGCGGCCGCCTAAGAAAACAATCGGGTGTTG	
Ltp1^{C10S}F	CCTATTCGTAAGTCTCGGCAAC	Generation of pcDNA3.1-Ltp1^{C10S}
Ltp1^{C10S}R	ATTTTATGTGGCTTCATTCAG	
